# Estrogen-Dependent Uterine Secretion of Osteopontin Activates Blastocyst Adhesion Competence

**DOI:** 10.1371/journal.pone.0048933

**Published:** 2012-11-09

**Authors:** Takashi Chaen, Toshihiro Konno, Mahiro Egashira, Rulan Bai, Nana Nomura, Shintaro Nomura, Yasushi Hirota, Toshihiro Sakurai, Kazuhiko Imakawa

**Affiliations:** 1 Laboratory of Animal Breeding, Department of Veterinary Medical Sciences, The University of Tokyo, Bunkyo-ku, Tokyo, Japan; 2 Department of Animal Bioscience, Nagahama Institute of Bio-Science and Technology, Nagahama, Shiga, Japan; 3 Department of Obstetrics and Gynecology, Faculty of Medicine, The University of Tokyo, Bunkyo-ku, Tokyo, Japan; State Key Laboratory of Reproductive Biology, Institute of Zoology, Chinese Academy of Sciences, China

## Abstract

Embryo implantation is a highly orchestrated process that involves blastocyst-uterine interactions. This process is confined to a defined interval during gestation referred to as the “window of embryo implantation receptivity”. In mice this receptive period is controlled by ovarian estrogen and involves a coordination of blastocyst adhesion competence and uterine receptivity. Mechanisms coordinating the acquisition of blastocyst adhesion competence and uterine receptivity are largely unknown. Here, we show that ovarian estrogen indirectly regulates blastocyst adhesion competence. Acquisition of blastocyst adhesion competence was attributed to integrin activation (e.g. formation of adhesion complexes) rather than de novo integrin synthesis. Osteopontin (OPN) was identified as an estrogen-dependent uterine endometrial gland secretory factor responsible for activating blastocyst adhesion competence. Increased adhesion complex assembly in OPN-treated blastocysts was mediated through focal adhesion kinase (FAK)- and phosphatidylinositol 3-kinase (PI3K)-dependent signaling pathways. These findings define for the first time specific regulatory components of an estrogen-dependent pathway coordinating blastocyst adhesion competence and uterine receptivity.

## Introduction

Implantation of the mammalian embryo into the uterus is a highly regulated process that involves apposition, attachment, and adhesion of blastocyst trophectoderm to uterine endometrial epithelium. In humans and rodents, this trophectoderm-endometrial epithelial interaction is followed by trophectoderm-derived trophoblast cell invasion into the endometrial stroma. Progression of this developmental program is dependent upon precise coordination of embryo and uterine endometrial competencies [1]. Acquisition of embryo and uterine endometrial competencies are confined to a specific interval during gestation referred to as the “window of embryo implantation receptivity” [2], [3].

In vitro blastocyst adhesion analyses have been used to gain a better understanding of the differentiation of non-adhesive trophectoderm into adhesive trophectoderm [3]. These efforts have resulted in the identification of a role for extracellular matrix (ECM) proteins in activating blastocyst adhesiveness. ECM proteins such as laminin, fibronectin (FN), and vitronectin (VTN) facilitate blastocyst adhesion and trophoblast outgrowth [3]. Moreover, soluble FN enhances blastocyst adhesive properties [4]. This cellular recognition event is mediated by the Arg-Gly-Asp (RGD) motif present in ECM proteins and acts through interactions with integrins situated on the trophectoderm cell surface [5], [6]. The identity of the RGD-bearing ligand responsible for blastocyst acquisition of adhesion competence has not been elucidated and may be complex due to the presence of several proteins at the implantation site with RGD motifs, such as FN, VTN and osteopontin (OPN) [7]–[9].

Coordination of blastocyst adhesion competence and uterine receptivity is dependent upon estrogen [1]. The means by which estrogen coordinates blastocyst adhesion competence during the window of embryo implantation receptivity is unknown. In this study, we investigated estrogen-activated mechanisms synchronizing the acquisition of blastocyst adhesion competence and uterine endometrial receptivity.

## Results

### Inhibition of Maternal Estrogen Action Impacts Blastocyst-fibronectin Adhesion Competence

**Figure 1:**
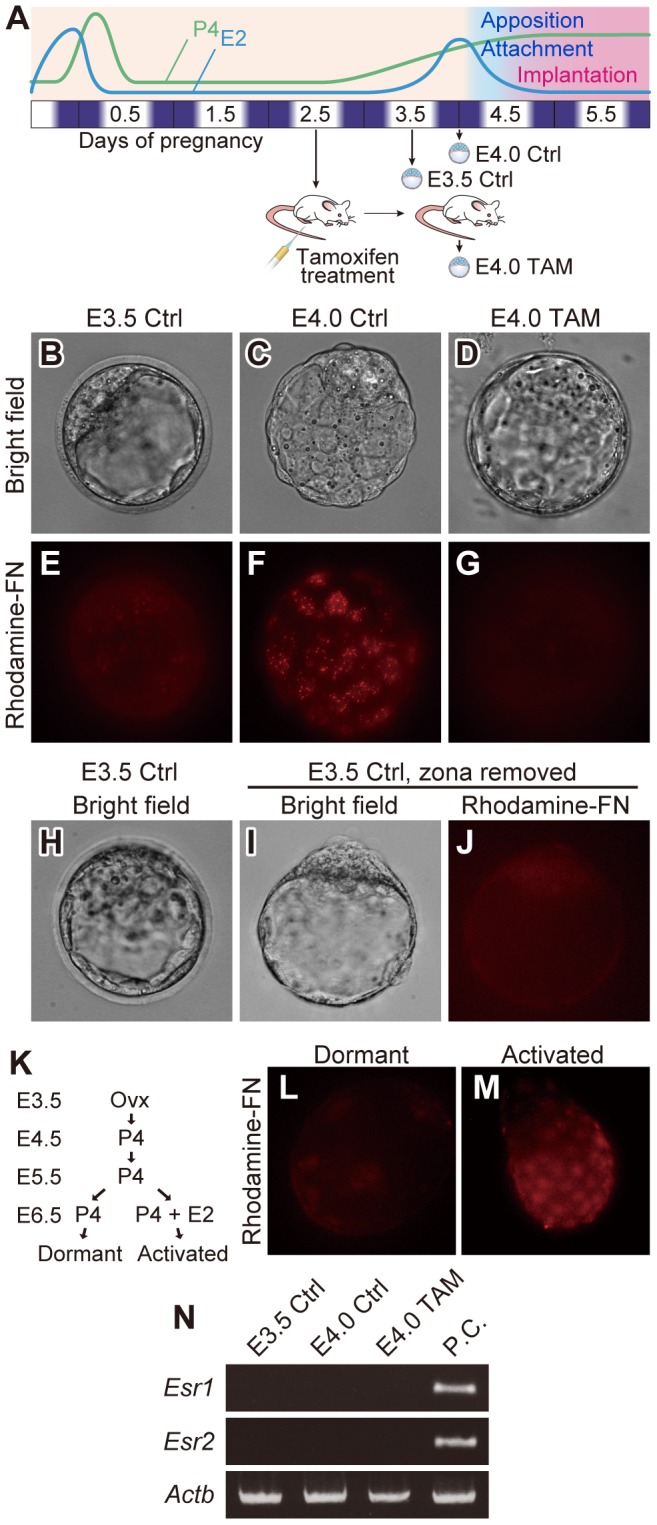
Maternal estrogen signaling impacts blastocyst adhesiveness. (***A***) Pregnant mice were treated on E2.5 with the estrogen competitor, tamoxifen. Blastocysts were collected from tamoxifen treated mice on E4.0 (E4.0 TAM) and control mice on E3.5 (E3.5 Ctrl) or E4.0 (E4.0 Ctrl) and rhodamine-FN binding assessed. (***B–G***) Blastocysts recovered from E3.5 Ctrl (***B*** **and** ***E***), E4.0 Ctrl (***C*** **and** ***F***) or E4.0 TAM (***D*** **and** ***G***) mice were incubated with rhodamine-conjugated FN (Rhodamine-FN) in DMEM/F12 medium. FN was monitored by rhodamine fluorescence (***E–G***). Bright field images of ***E–G*** are presented in ***B–D***. Only hatched blastocysts at E4.0 were examined. (***H–J***) Blastocysts collected from E3.5 mice (***H***) were treated with acid Tyrode’s solution to remove zona pellucida (***I***) and subjected for rhodamine-FN binding assay (***J***). (***B–J***) Multiple samples were analyzed on at least three separate occasions. Representative results are presented. (***K***) Schematic representation of procedure to generate dormant and activated blastocysts. Mice were ovariectomized on the morning of E3.5. Blastocyst dormancy in delayed implantation-mice was maintained by daily injection of progesterone from E3.5–6.5. To activate dormant blastocysts, delayed implantation-mice were injected with 17β-estradiol on E6.5. Dormant or activated blastocysts were collected 10 hours after the last steroid injections. (***L*** **and** ***M***) Dormant or activated blastocysts were examined for their adhesiveness by rhodamine-FN binding assay. Multiple samples were analyzed on at least three separate occasions. Representative results are presented. (***N***) RT-PCR analyses of estrogen receptors in blastocysts. Transcripts of *Esr1* and *Esr2* by which estrogen receptors alpha and beta are encoded, respectively, were not detected in the blastocysts. Total RNAs from three pools of 30 blastocysts for each groups were analyzed. *Actb* served as a control for the RNA integrity.

In mice, the uterine “window of embryo implantation receptivity” is controlled by estrogen [10]. Embryo implantation can be prevented by removal of ovarian estrogen [11] and interference of estrogen interactions with its receptor [12]. We evaluated the effect of disrupting estrogen receptor signaling on blastocyst adhesiveness (**Fig. 1** ***A***). Pregnant mice were treated with an estrogen competitor, tamoxifen (TAM) [12], [13], on E2.5 and blastocysts were collected on E4.0 (E4.0 TAM). Blastocysts were also collected from intact pregnant mice on E3.5 or E4.0 (E3.5 Ctrl and E4.0 Ctrl, respectively) and examined for their affinity for FN using a rhodamine-FN binding assay. Blastocysts collected from intact mice on E4.0 showed strong binding to FN (**Fig. 1** **** ***C*** **and** ***F***), while E3.5 Ctrl blastocyst binding to FN was negligible (**Fig. 1** **** ***B*** **and** ***E***) even after zona removal (**Fig. 1** **** ***H–J***). E4.0 blastocysts from TAM-treated mice did not bind FN (**Fig. 1** **** ***D*** **and** ***G***), indicating that estrogen was essential for acquisition of blastocyst-FN adhesion competence. The effect of maternal estrogen on blastocyst adhesiveness was also observed in the mouse delayed implantation model (**Fig. 1** **** ***K–M*** **)**. Dormant blastocysts collected from mice in diapause exhibited limited binding to FN (**Fig. 1** ***L***), which was dramatically upregulated by maternal supplementation with 17β-estradiol (**Fig. 1** ***M***). Although blastocyst-FN adhesion appeared to be estrogen dependent, mRNAs that encode estrogen receptor alpha and beta were not detected on the blastocyst (**Fig. 1** ***N***). The results indicate that the blastocyst-FN binding is controlled by estrogen; however, the actions of estrogen on blastocyst-FN adhesion are probably indirect.

### Uterine Receptivity Influences Adhesion Complex Assembly on Trophectoderm

**Figure 2:**
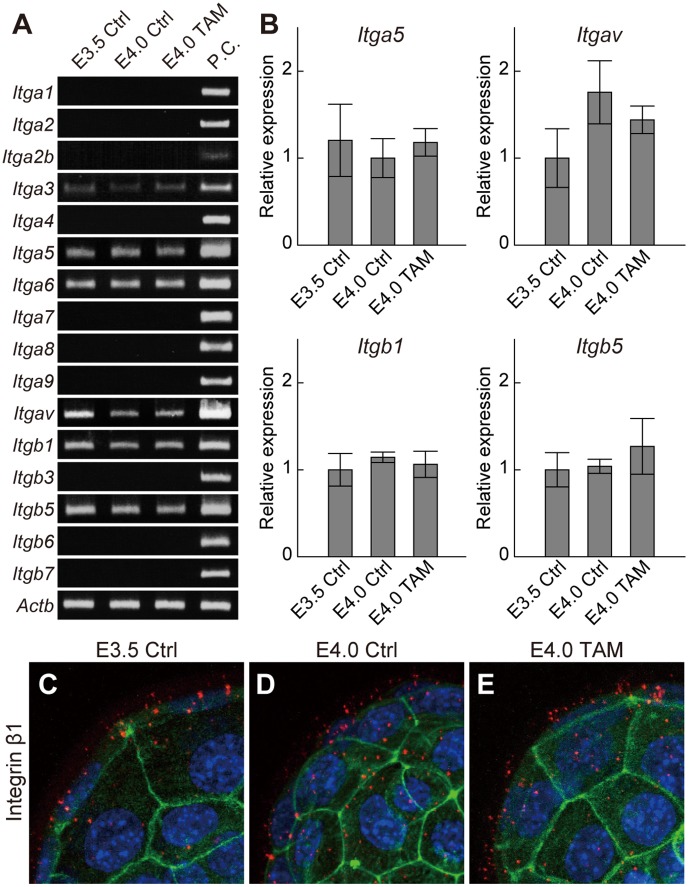
Effects of maternal estrogen signaling on blastocyst integrin expression. (***A***) Expression of ECM-binding type integrins in E3.5 Ctrl, E4.0 Ctrl and E4.0 TAM blastocysts were examined by RT-PCR (three pools of 30 blastocysts for each group). P.C., positive control. (***B***) Transcription levels of FN- and OPN-binding type integrins were quantified. qRT-PCR for *Itga5*, *Itgav*, *Itgb1* and *Itgb5* in E3.5 Ctrl, E4.0 Ctrl and E4.0 TAM blastocysts (three pools of 30 blastocysts for each group) were evaluated by analysis of variance. Data are presented as mean±SEM. (***C–H***) Immunofluorescence staining for integrin β1 in E3.5 Ctrl, E4.0 Ctrl and E4.0 TAM blastocysts. Integrin β1 was labeled with Alexa 568 (red). F-actin and nuclei were stained with phalloidin-Atto 488 (green) and DAPI (blue), respectively. Z-stack projection images of confocal laser-scanning microscopy are presented. Multiple samples were analyzed on at least three separate occasions. Representative results are presented.

Estrogen promotes uterine receptivity [14]. Cells interact with FN and other ECM components through the assembly of heterodimeric proteins, termed integrins [15]. We next examined the effect of nidatory estrogen on blastocyst expression of integrins. Blastocysts expressed α3, α5, α6, αV, β1 and β5 integrins (**Fig. 2** ***A***). Of these integrin subunits, α5, αV, β1 and β5 are known to assemble into effective binding partners for FN and other RGD-bearing ligands such as VTN and OPN [15]. Transcript levels for this subset of integrin subunits were not affected by maternal estrogen signaling (**Fig. 2** ***B***). Blastocyst integrin β1 subunit protein distribution was also not affected by maternal estrogen signaling (**Fig. 2** **** ***C–E***). These observations indicate that the estrogen/uterine receptivity-dependent activation of blastocyst adhesion to FN was not the consequence of de novo integrin synthesis.

**Figure 3:**
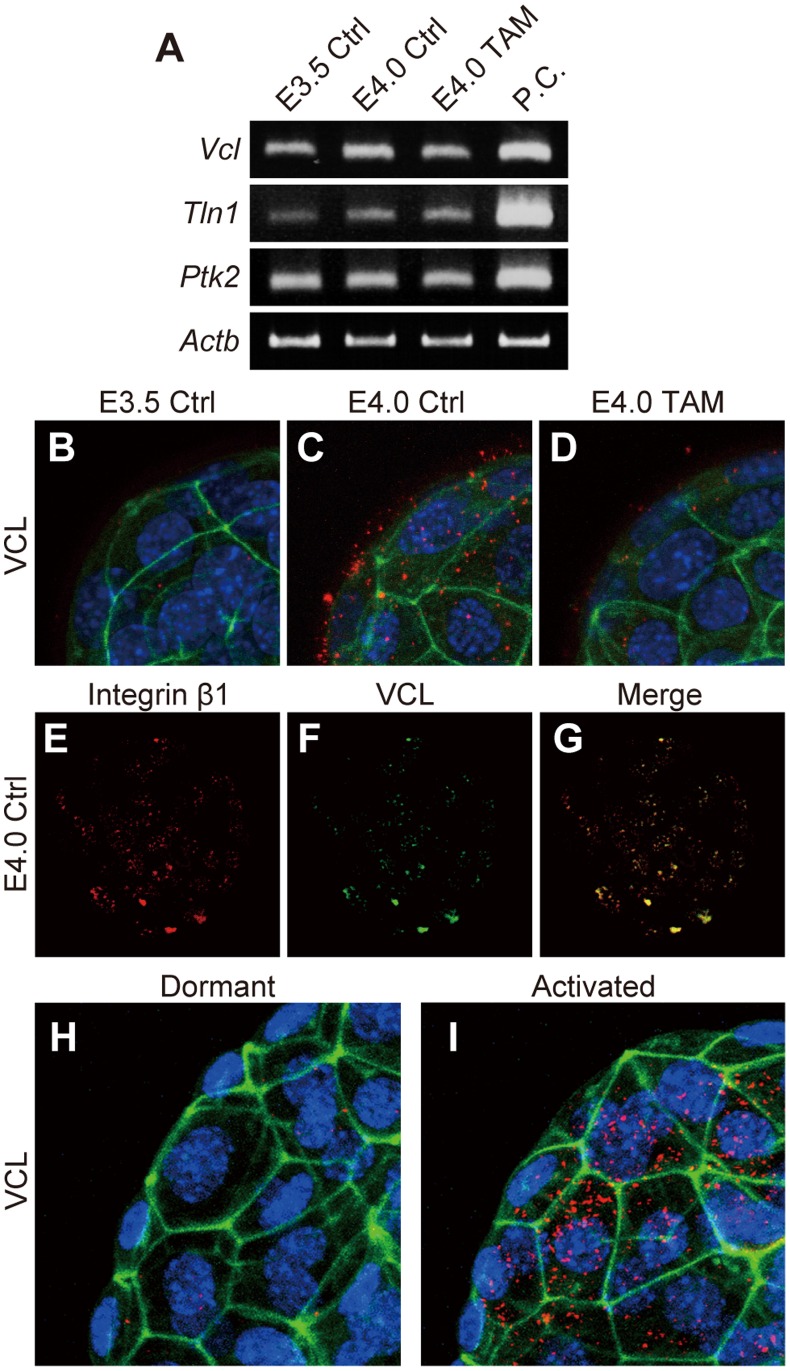
Maternal estrogen signaling induces adhesion complex assembly in the trophectoderm (*A*) RT-PCR analyses of integrin adhesion complex components. Transcripts of *Vcl, Tln1* and *Ptk2* that encode vinculin, talin and focal adhesion kinase, respectively, were expressed in blastocysts, however expression levels of these transcripts were not affected by the uterine receptivity. Total RNAs from three pools of 30 blastocysts for each groups were analyzed and representative results presented. (***B–D***) Immunofluorescence staining for VCL in E3.5 Ctrl, E4.0 Ctrl and E4.0 TAM blastocysts. VCL was labeled with Alexa 568 (red). F-actin and nuclei were stained with phalloidin-Atto 488 (green) and DAPI (blue), respectively. (***E–G***) Dual-immunofluorescence staining for integrin β1 and VCL was performed on E4.0 Ctrl blastocysts. Integrin β1 (***E***) and VCL (***F***) were labeled with Alexa 568 and FITC, respectively. Yellow signals in merged image (***G***) indicate co-localization of integrin β1 and VCL. (***H*** **and** ***I***) Immunolocalization of VCL in dormant or activated blastocysts. VCL proteins were labeled with Alexa 568 (red). F-actin and nuclei were stained with phalloidin-Atto 488 (green) and DAPI (blue), respectively. The signals of VCL were found at the surface of activated blastocyst (***I***), whereas that was hardly detectable in the dormant blastocysts (***H***). (***B–I***) Z-stack projection images of confocal laser-scanning microscopy are presented. Multiple samples were analyzed on at least three separate occasions. Representative results are presented.

The assembly of integrin heterodimers occurs at the cell surface where they exist as inactive or functional receptors [16]. Functional integrin signaling requires the formation of a functional integrin adhesion complex with other regulatory proteins such as VCL, talin (TLN1), and focal adhesion kinase (PTK2) [17]. *Vcl*, *Tln1*, and *Ptk2* mRNAs were expressed by blastocysts but their levels were not affected by uterine receptivity (**Fig. 3** ***A***). However, VCL protein distribution was affected by estrogen (**Fig. 3** **** ***B–D***). At E3.5 VCL protein was difficult to detect (**Fig. 3** ***B***), whereas at E4.0 VCL protein accumulated at the trophectoderm surface of blastocysts (**Fig. 3** ***C***). VCL protein expression was also negligible in E4.0 blastocysts recovered from TAM treated pregnant female mice (**Fig. 2** ***D***). Cell surface VCL co-localized with integrin β1 (**Fig. 3** **** ***E–G***), suggesting formation of the adhesion complex. Maternal 17β-estradiol supplementation also stimulated VCL protein distribution in diapaused blastocysts (**Fig. 3** **** ***H*** **and** ***I***). These results indicate that the estrogen-primed uterus induces adhesion complex assembly in the trophectoderm, which results in uterine receptivity-dependent activation of blastocyst adhesiveness.

### OPN Expression in Estrogen-stimulated Endometrial Glandular Epithelium

**Figure 4:**
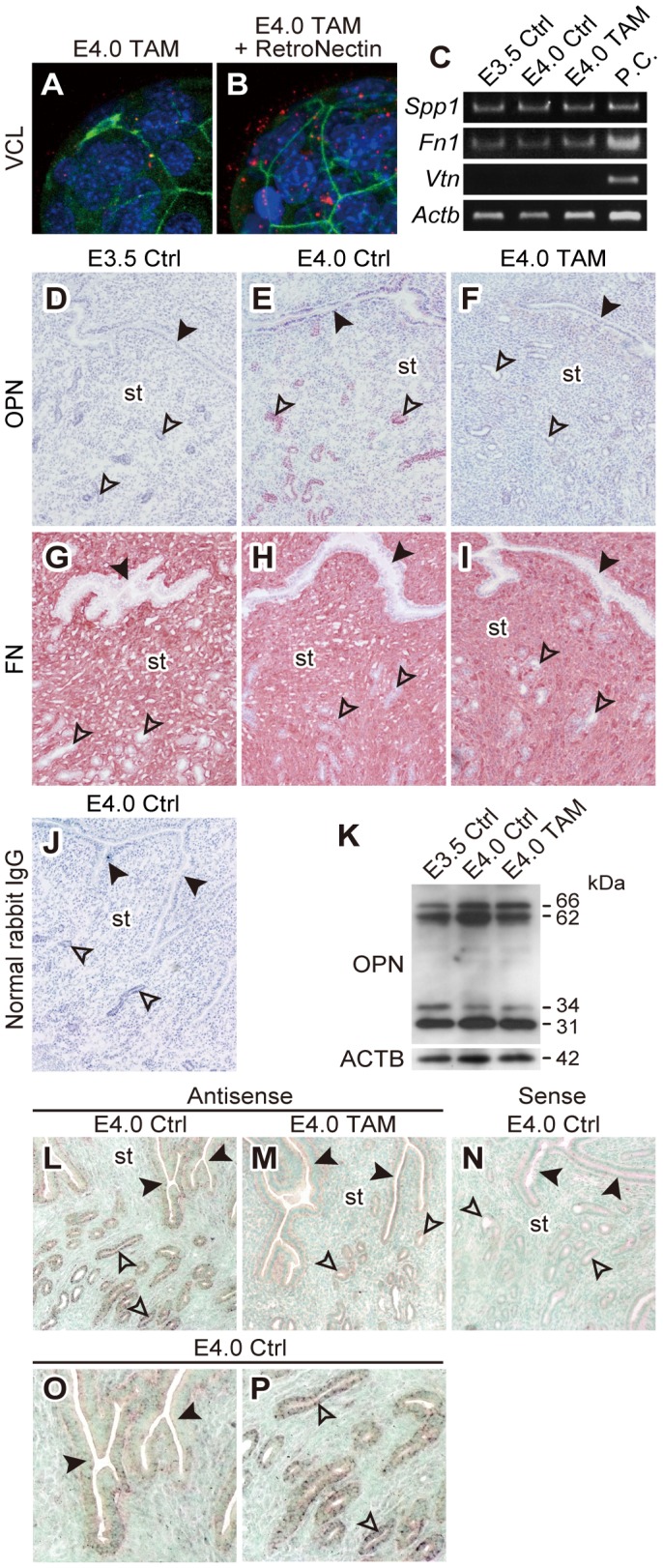
Endometrial glandular epithelial cells from the receptive uterus express osteopontin. (***A*** **and** ***B***) Blastocysts collected from E4.0 TAM mice were incubated for 90 min with or without RetroNectin in DMEM/F12 medium. The effect of RetroNectin treatment on expression and distribution of VCL were then evaluated by immunofluorescence. Immunolocalization of VCL was visualized with Alexa 588 (red). F-actin and nuclei were stained with phalloidin-Atto 488 (green) and DAPI (blue), respectively. Z-stack projection images of confocal laser-scanning microscopy are presented. Multiple samples were analyzed on at least three separate occasions. (***C***) Expression of *Spp1*, *Fn1* and *Vtn* mRNAs in E3.5 Ctrl, E4.0 Ctrl and E4.0 TAM uteri were evaluated by RT-PCR. Total RNAs from three uteri for each group were analyzed. P.C., positive control. (***D–J***) Immunohistochemistry for OPN (***D–F***) and FN (***G–I***) was performed on uterine tissues from E3.5 Ctrl, E4.0 Ctrl or E4.0 TAM mice. Normal rabbit IgG served as a negative control (***J***). Uterine tissues from at least three animals for each group were analyzed. Representative results are presented. Black arrowheads: luminal epithelium, open arrowheads: glandular epithelium, st: stroma. (***K***) Western blotting for OPN in protein extracts from E3.5 Ctrl, E4.0 Ctrl and E4.0 TAM uteri. Uterine tissues from at least three animals for each group were analyzed. Representative blot is presented. β-actin was used as loading control. Four species of immunoreactive OPN were found at molecular weights of approximately 66 kDa, 62 kDa, 34 kDa and 31 kDa. (***L–P***) In situ hybridization for *Spp1* transcripts on E4.0 uterine tissues. Antisense (***L*** **and** ***M***) and sense (***N***) probes for *Spp1* mRNA were hybridized on uterine tissues from E4.0 Ctrl (***L*** **and** ***N***) and E4.0 TAM (***M***) mice. (***O*** **and** ***P***) Higher magnification images of ***L*** highlighting luminal epithelia (***O***) and glandular epithelia (***P***) of E4.0 Ctrl uterus. Note that the expression of *Spp1* mRNA on E4.0 uterus (***L***) was more prominent in glandular epithelia (***P***) than that in luminal epithelia (***O***) and disrupted by TAM treatment (***M***). Sense probe did not show any signals on E4.0 uterus (***N***). Uterine tissues from at least three animals for each group were analyzed. Representative results are presented. Tissue sections were counter-stained with methyl green. Black arrowheads: luminal epithelium, open arrowheads: glandular epithelium, st: stroma.

Our results demonstrated that blastocyst adhesiveness (formation of integrin adhesion complexes) was influenced by uterine receptivity. However, the molecular signal from the estrogen-primed uterus responsible for initiating the formation of integrin adhesion complexes is unknown. Previous in vitro studies have shown that FN facilitates blastocyst adhesion and outgrowth [3]. This process may be mediated through interactions with extracellular proteins possessing RGD motifs [5]. In order to evaluate whether RGD-containing extracellular proteins induce adhesion complex formation at the blastocyst surface, E4.0 TAM blastocysts were incubated with RetroNectin, a recombinant protein containing RGD domain. Immunofluorescence analyses demonstrated that VCL molecules accumulated at the surface of blastocysts after ex vivo treatment with RetroNectin (**Fig. 4** ***A*** **and** ***B***), indicating that the RGD containing extracellular proteins could act as a cue to induce adhesion complex formation in the blastocyst.

**Figure 5:**
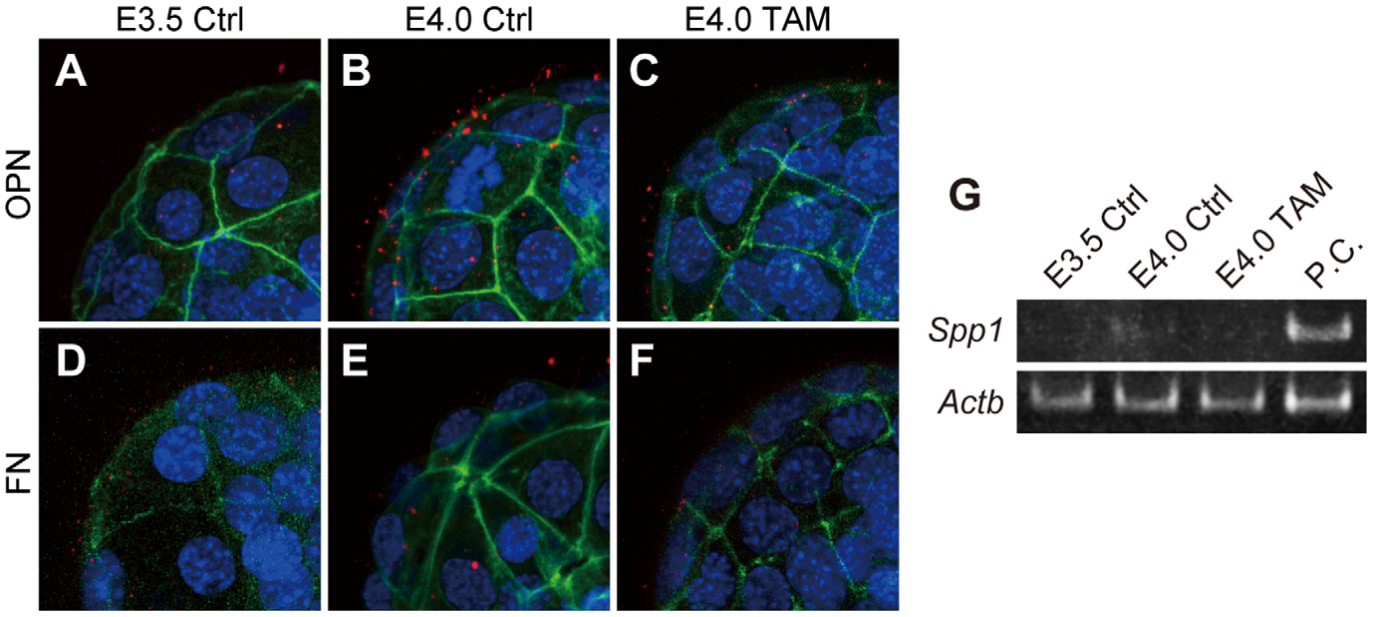
Endometrial gland derived OPN interacts with blastocysts. Immunofluorescence staining for OPN (***A–C***) and FN (***D–F***) was performed on E3.5 Ctrl, E4.0 Ctrl and E4.0 TAM blastocysts. OPN and FN were labeled with Alexa 568 (red). F-actin and nuclei were stained with phalloidin-Atto 488 (green) and DAPI (blue), respectively. Z-stack projection images of confocal laser-scanning microscopy are presented. Multiple samples were analyzed on at least three separate occasions. (***G***) RT-PCR analysis of *Spp1* in blastocysts. Transcript of *Spp1* that encodes OPN was not detected in the blastocysts. Total RNAs from three pools of 30 blastocysts for each group were analyzed and representative results presented. P.C., positive control.

**Figure 6:**
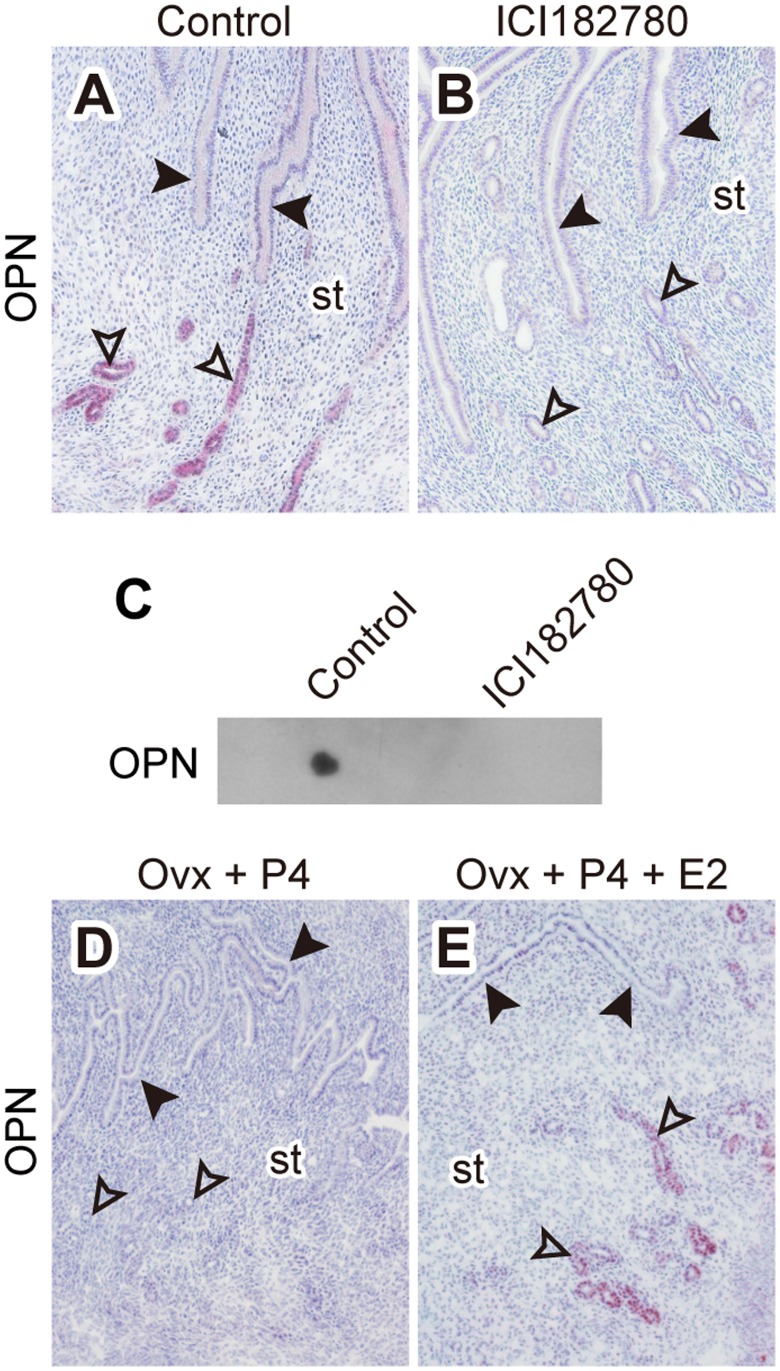
OPN is secreted from endometrial glands in an estrogen-dependent manner. (***A–C***) Pregnant mice were treated with ICI 182,780 (0.5 mg/mouse, s.c. injection) on E2.5. Uterine tissues were collected from control (***A***) and ICI 182,780 treated (***B***) mice on E4.0 and examined for OPN expression by immunohistochemistry. Multiple samples were analyzed and representative results presented. Black arrowheads: luminal epithelium, open arrowheads: glandular epithelium, st: stroma. (***C***) Uterine flashing media from E4.0 control and ICI 182,780 treated mice were examined for the presence of OPN by dot blot analysis. E4.0 uteri from control or ICI 182,780 treated mice were flushed with 100 µl of PBS. 2 µl of each flushing medium was subjected to dot blotting for OPN. Multiple samples were analyzed and representative results presented. (***D*** **and** ***E***) Immunohistochemistry for OPN on uterine tissues from delayed implantation-mice. Endometrial glandular expression of OPN was not detected in the uterus from the progesterone-maintained delayed implantation-mice (***D***) and induced by supplementation of 17β-estradiol (***E***). Uterine tissues from at least three animals from each group were analyzed and representative results presented. Black arrowheads: luminal epithelium, open arrowheads: glandular epithelium, st: stroma.

We next investigated the uterine expression of RGD containing ECM proteins, including FN, VTN, and OPN. RT-PCR analyses of uteri from E3.5 Ctrl, E4.0 Ctrl and E4.0 TAM demonstrated the expression of *Fn1* and *Spp1* mRNAs, which encode FN and OPN respectively, whereas *Vtn* mRNA, which encodes VTN, was not detected (**Fig. 4** ***C***). Immunohistochemistry of peri-implantation uteri showed that OPN was expressed in glandular epithelia of E4.0 Ctrl uteri (**Fig. 4** ***E***), whereas the glandular expression of OPN was not observed in E3.5 Ctrl or E4.0 TAM treated uteri (**Fig. 4** **** ***D*** **and** ***F***). FN was abundantly expressed throughout the uterine stroma, however, the expression was not observed in either luminal or glandular epithelia (**Fig. 4** **** ***G–I***). Although the immunohistochemistry demonstrated estrogen-dependency in glandular OPN expression (**Fig. 4** **** ***D–F***), estrogen did not appear to affect uterine OPN transcript levels, as measured by RT-PCR (**Fig. 4** ***C***), and protein levels, as detected by western blotting (**Fig. 4** ***K***). These quantitative measurements might be confounded by OPN expression in circulating mononuclear cells within the uterus [18]. In situ localization for *Spp1* transcripts on E4.0 uteri demonstrated the expression of *Spp1* primarily in glandular epithelia, though weak expression was also found in luminal epithelia (**Fig. 4** ***L***). Expression in both epithelia was disrupted by TAM treatment (**Fig. 4** ***M***). These results raise the possibility that OPN, rather than the FN, is secreted from the estrogen-primed endometrium and contributes to the activation of blastocyst adhesiveness. Therefore, we next attempted to detect OPN and FN proteins at the blastocyst surface by immunofluorescence. OPN was found on the surface of blastocysts in a maternal estrogen-dependent manner (**Fig. 5** **** ***A–C***), whereas FN was difficult to detect at the surface of any of the three types of blastocysts (**Fig. 5** **** ***D–F***). Furthermore, *Spp1* mRNA was not detected in the blastocysts (**Fig. 5** ***G***) indicating that the OPN found at the blastocyst surface was of endometrial origin. In order to further evaluate the estrogen-dependency of uterine glandular OPN expression/secretion, we next treated pregnant mice with ICI 182,780 (ICI), a pure estrogen receptor antagonist, on E2.5 [19]. Uterine glandular OPN expression on E4.0 was efficiently inhibited by ICI treatment (**Fig. 6** **** ***A*** **and** ***B***). Dot blot analysis of uterine flushing media demonstrated the presence of OPN in uterine lumen of E4.0 Ctrl mice; whereas OPN was not detected in flushing media from ICI treated E4.0 uteri (**Fig. 6** ***C***). We also determined that the OPN was not expressed in uterine glandular epithelium of mice in diapause (**Fig. 6** ***D***) but was induced following 17β-estradiol treatment (**Fig. 6** ***E***). The results suggest that the estrogen-dependent secretion of OPN by glandular epithelium of the receptive uterus acts as an extracellular signal to initiate activation of blastocyst adhesiveness.

### OPN Activation of Blastocyst Adhesion Complex Assembly

**Figure 7:**
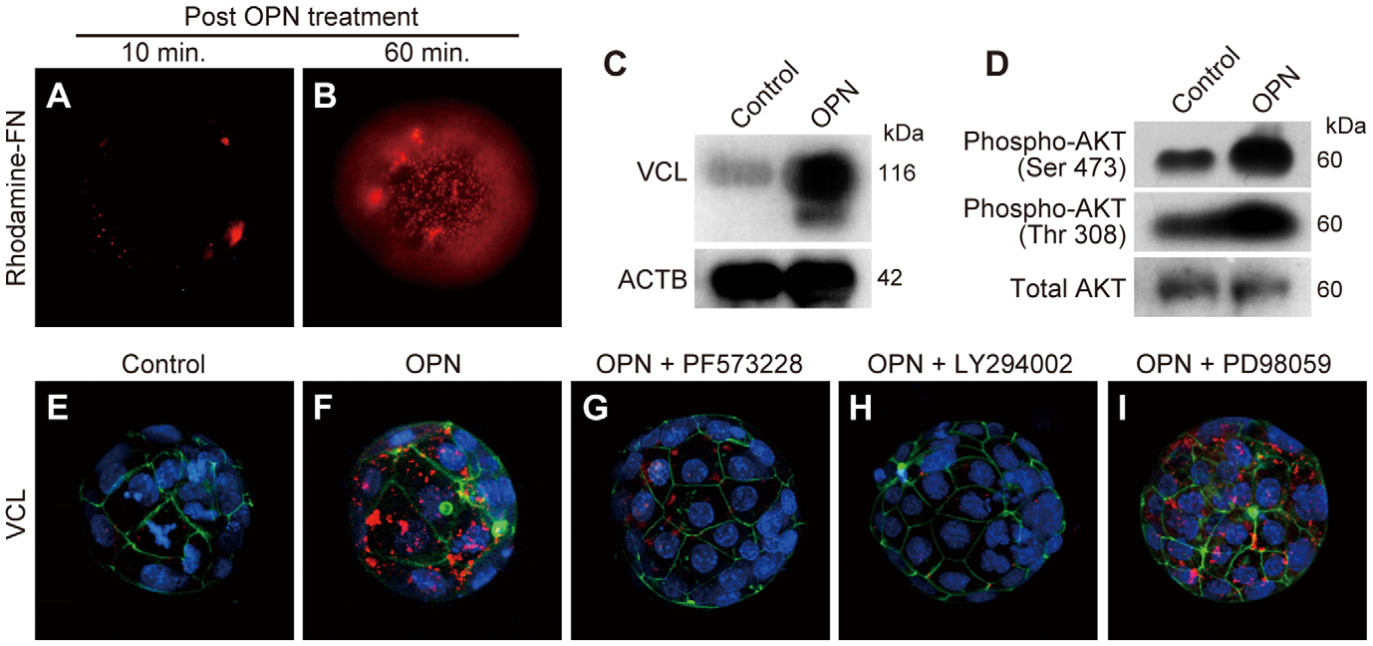
Osteopontin-induced blastocyst adhesiveness is mediated through FAK/PI3K signaling. (***A*** **and** ***B***) IVF-derived blastocysts were incubated with OPN in DMEM/F12 media for 10 min then transferred to culture medium without OPN. The effect of OPN-priming on blastocyst adhesiveness was monitored for rhodamine-FN binding at 10 min (***A***) and 60 min (***B***) after OPN treatment. (***C*** **and** ***D***) IVF-derived blastocysts were incubated with or without OPN in DMEM/F12 media for 90 min and examined for VCL expression (***C***) and AKT phosphorylation (***D***) by immunoblotting. Three pools of ten blastocysts for each control group (Control) and OPN treated group (OPN) were analyzed. β-actin (ACTB) and total AKT were used as loading controls for ***C*** and ***D***, respectively. (***E*** **and** ***F***) IVF-derived blastocysts were incubated with or without OPN in DMEM/F12 for 90 min and examined for expression and distribution of VCL by immunofluorescence staining. (***G–I***) Effects of small molecule inhibitors for FAK (***G***), PI3K (***H***) or MEK1/2 (***I***) on the expression and distribution of VCL during OPN-induced blastocyst adhesiveness were examined by VCL immunofluorescence staining. (***E–I***) Immunolocalization of VCL was visualized with Allexa 568 (red). F-actin and nuclei were stained with phalloidin-Atto 488 (green) and DAPI (blue), respectively. Z-stack projection images of confocal laser-scanning microscopy are presented. (***A*** **,** ***B*** **and** ***E*** **–** ***I***) Multiple samples were analyzed on at least three separate occasions. Representative results are presented.

Our results demonstrated that OPN is secreted from endometrial glands of the estrogen-primed receptive uterus. To evaluate whether OPN could activate blastocyst adhesiveness, we produced blastocysts by in vitro fertilization (IVF) and in vitro culture. IVF-derived blastocysts were incubated with OPN for 10 min, followed by 10 or 60 min incubation in DMEM/F12 to allow formation of adhesion complexes. The effects of OPN-priming on blastocyst adhesiveness were then assessed by rhodamine-FN binding. OPN increased blastocyst binding to rhodamine-FN (**Fig. 7** ***A*** **and** ***B***). FN-binding was most prominent 60 min after OPN treatment.

OPN binding to its cell surface receptors, including CD44 and integrins, initiates a variety of kinase cascades, including FAK and PI3K/AKT signaling [18], [20], [21]. OPN treatment resulted in increased VCL protein expression (**Fig. 7** ***C***) and accumulation at the blastocyst surface (**Fig. 7** **** ***E*** **and** ***F***), indicating that the formation of adhesion complexes is induced by OPN-receptor interaction. Blastocyst AKT phosphorylation also increased following OPN treatment (**Fig. 7** ***D***). The results raise the possibility that the formation of integrin adhesion complexes in the blastocyst is mediated through PI3K activation following OPN-receptor interaction. Thus, we next examined the effect of small molecule inhibitors for PI3K (LY294002), FAK (PF573228), and MEK1/2 (PD98059) on the formation of adhesion complexes activated by OPN treatment of IVF-derived blastocysts. OPN-stimulated VCL expression and blastocyst surface localization were disrupted by inhibition of PI3K or FAK (**Fig. 7** **** ***G*** **and** ***H***). However, the inhibition of MEK1/2 (an activator of MAPK signaling) had no effect on OPN-induced assembly of blastocyst adhesion complexes (**Fig. 7** ***I***). These observations indicate that the OPN activation of blastocyst adhesiveness is mediated through FAK- and PI3K-dependent signaling pathways.

Collectively, the results indicate that the blastocyst acquires adhesion competence by forming integrin adhesion complexes at the blastocyst surface. Estrogen-dependent uterine glandular OPN expression/secretion acts as an extracellular signal inducing blastocyst adhesion complex assembly. OPN-induced blastocyst adhesion complex assembly is mediated through FAK and PI3K signaling.

## Discussion

**Figure 8:**
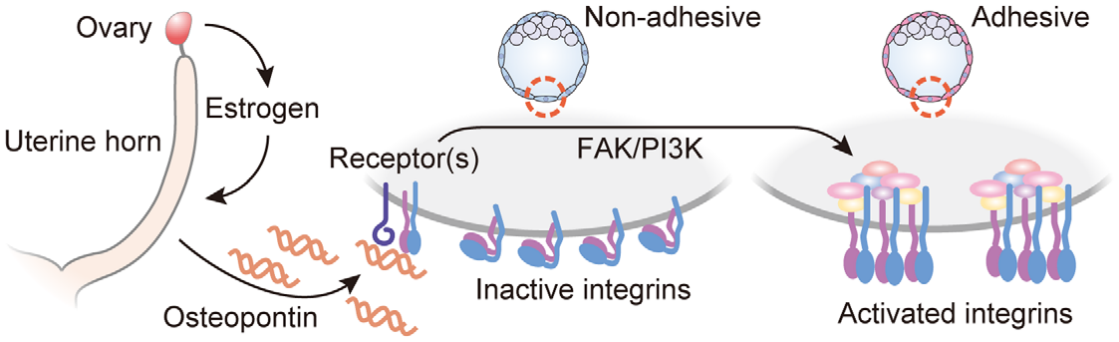
Proposed model of blastocyst activation of adhesiveness during implantation process. Acute increase of serum estrogen, which controls uterine “window of receptivity,” stimulates endometrial glandular expression of OPN. OPN binding to blastocyst surface receptors (CD44 and/or integrins) initiates FAK- and PI3K-dependent signaling cascade by which integrins are activated. This estrogen-dependent activation of blastocyst integrins ensures conformity between blastocyst adhesiveness and uterine receptivity.

Estrogen is a key hormone coordinating disparate events within the uterus and the embryo required for embryo implantation. Central to the synchronization between the uterus and embryo is OPN. OPN is secreted from uterine endometrial glands in an estrogen-dependent manner and acts on the embryo to promote blastocyst adhesion competence (**Fig. 8**). Blastocysts quickly activate their adhesion competence in response to OPN via formation of integrin adhesion complexes at the trophectoderm cell surface.

In mice, integrin subunits α5, α6 and β1 are known to be expressed throughout early embryo development [22]. Combinations of these subunits assemble into integrins capable of interacting with RGD containing ligands (α5β1) and laminin (α6β1) [15]. In the present study, we identified the expression of several RGD binding integrins (α5β1, αvβ1 and αvβ5) in blastocysts. Human and rabbit blastocysts have also been shown to express αvβ3, which is an integrin capable of binding RGD containing ligands [23], [24]. Integrin expression profiles did not correlate with blastocyst adhesion competence. Translocation of integrins to the trophectodermal cell surface of the blastocyst is required for blastocyst adhesion [25] but is not sufficient to promote adhesion competence until blastocysts are exposed to RGD bearing ligands [26], suggesting the involvement of another mechanism in the activation of blastocyst adhesion competence. VCL, a cytoplasmic actin-binding protein, plays a critical role in clustering integrin heterodimers into oligomers facilitating their assembly into functional integrin adhesion complexes, which promote cell-extracellular matrix interactions and transmit integrin-ligand signals to cytoplasmic signaling transducers [27]. This integrin “outside-in” signaling can influence a variety of cellular biological events, including cellular adhesion, migration, survival and differentiation [17].

OPN is a member of the small integrin-binding ligand N-linked glycoprotein (SIBLING) family, which are all characterized by RGD integrin binding tripeptide and N-linked oligosaccharide motifs [28]. OPN is also capable of interacting with the cell surface glycoprotein, CD44, which is involved in cell adhesion [18]. Although the SIBLINGs are prominent in mineralized tissues, they are distributed in a limited range of tissues and are highly expressed in many cancers [28]. OPN is predominantly a secretory protein found in body fluids [29]. Secreted OPN exists as both a soluble cytokine and an immobilized protein adsorbed to calcified matrices. OPN contributes to many biological processes, including cellular adhesion, migration and survival [30] and has been implicated in embryo implantation and placentation processes in a range of mammalian species [31]. In mice, it has previously been reported that uterine luminal epithelium expresses OPN in an estrogen-dependent manner [32]. Our results also demonstrated the estrogen-dependent OPN expression, however, the expression was more prominent in uterine gland, consistent with nidatory estrogen inducing OPN secretion from uterine glands. The respective involvement of integrins and CD44 in OPN activated blastocyst adhesion competence requires further investigation. We report for the first time that OPN is capable of activating blastocyst adhesion competence.

OPN-blastocyst interactions activated intracellular signaling pathways impacting AKT serine/threonine protein kinase. AKT is a major signal transducer downstream of PI3K and has a wide range of substrates involved in many cellular processes [33]. The PI3K/AKT signaling pathway has been implicated as a potential regulator of trophoblast invasion. PI3K/AKT becomes constitutively activated upon differentiation of trophoblast stem cells and regulates a set of trophoblast cell differentiation-dependent genes encoding proteins contributing to the invasive and endocrine phenotypes of trophoblast giant cells [34], [35]. PI3K/AKT signaling can also be activated by FAK [36]. Inhibition of either PI3K or FAK results in disruption of OPN-induced blastocyst adhesion complex assembly, indicating that the OPN-dependent acquisition of blastocyst adhesion competence is mediated through FAK- and PI3K-dependent pathways. Although, OPN is capable of activating blastocyst adhesiveness, OPN is not essential for reproduction in the mouse. Mice with a null mutation within the gene encoding OPN, *Spp1,* are fertile [9]. Therefore, other uterine-derived factors that can bind integrins and/or CD44 may compensate for OPN activation of blastocyst adhesion competence. There are other key regulatory proteins produced by the endometrial glandular epithelium in an estrogen-dependent manner (e.g. leukemia inhibitory factor: LIF) that are critical for embryo implantation [37], [38]. LIF mediates uterine expression of several proteins that are implicated in the uterine receptivity, such as cyclooxygenase-2 and several epidermal growth factor family cytokines [39]. LIF and heparin-binding EGF-like growth factor could participate, at least in part, in the acquisition of blastocyst adhesion competence through activating PI3K [40], [41].

In conclusion, estrogen coordinates both uterine receptivity and blastocyst adhesion competence. These actions on the uterus and embryo are both direct and indirect. Adhesion competence is acquired through the actions of estrogen on endometrial gland OPN secretion. OPN then acts on the trophectoderm cell surface to activate FAK and PI3K/AKT signaling pathways, which regulate the formation of functional integrin adhesion complexes.

## Materials and Methods

### Animals and Tissue Preparation

ICR mice (CREA Japan, Tokyo, Japan) were housed in an environmentally controlled facility and allowed free access to food and water. Timed matings of animals were conducted by placing females (>8 weeks of age) with fertile males. The day when a seminal plug was found in the vagina of female mice was defined as gestation day (E) 0.5. Pregnant mice were intraperitoneally (i.p.) injected with tamoxifen citrate (10 µg per mouse, Wako, Osaka, Japan) on E2.5. Mice were killed on E3.5 (morning of E3.5) or E4.0 (midnight of E3.5) and uterine tissues dissected. Uterine tissues were either snap-frozen in liquid nitrogen for RNA analysis or frozen in dry ice-cooled heptane for immunohistochemical analysis. Blastocysts were collected by flushing uteri with PBS or DMEM/F12 media and then subjected for further analyses. All of the procedures involving animals were reviewed and approved by the University of Tokyo Institutional Animal Care and Use Committee and were performed in accordance with the Guiding Principles for the Care and Use of Laboratory Animals.

### Delayed Implantation Model

To induce delayed implantation, pregnant mice were ovariectomized on the morning of E3.5 and subcutaneously (s.c.) injected daily with progesterone (2 mg per mouse, Sigma-Aldrich, St. Louis, MO) in the morning from E3.5 to E6.5. Dormant blastocysts were then recovered from delayed implantation-mice on E7.0. To obtain activated blastocysts, delayed implantation-mice were s.c. injected with 17β-estradiol (25 ng per mouse, Sigma-Aldrich) on E6.5. Blastocysts were collected 10 h after the last steroid injections. Uterine tissues from these mice were also collected and used for immunohistochemistry analyses.

### In vitro Fertilization (IVF) and Embryo Culture

Female mice were superovulated by treatment with PMSG (5 IU i.p. per mouse, ASKA Pharmaceutical, Tokyo, Japan) and 48 h later hCG (5 IU i.p. per mouse, ASKA Pharmaceutical). Females were sacrificed between 15–16 h following administration of hCG injection and oocytes collected. Epididymal sperm were collected from fertile males and activated in TYH medium. Oocytes were fertilized with the activated sperm in TYH medium, then cultured to blastocyst stage in KSOM medium.

### Rhodamine-FN Binding Assay

Blastocysts were briefly washed with PBS containing BSA and then incubated with rhodamine-conjugated FN (30 µg/ml; Cytoskeleton, Denver, CO) in DMEM/F12 medium at 37°C under 5% CO_2_ gas for 10 min. Blastocyst-FN binding was then immediately monitored by the rhodamine fluorescence under inverted fluorescence microscope (IX70, Olympus, Tokyo, Japan).

### Ex vivo Induction of Blastocyst Adhesiveness

Blastocysts obtained from tamoxifen treated mice were briefly washed in PBS and incubated with RetroNectin (100 µg/ml; Takara Bio Inc., Otsu, Japan) in DMEM/F12 media at 37°C for 90 min. RetroNectin-treated blastocysts were examined for expression and distribution of vinculin (VCL) by immunofluorescence. To evaluate the effect of OPN on blastocyst adhesiveness, IVF derived blastocysts were incubated with recombinant full length human OPN (2.5 µg/ml; catalog no. ab81549; Abcam, Cambridge, UK) in DMEM/F12 media for 10 min, and then transferred into DMEM/F12 medium without OPN. Blastocyst adhesiveness was then monitored at 10 or 60 min by assessing rhodamine-FN binding. IVF derived blastocysts were also incubated in OPN containing medium for 90 min and then examined for VCL expression and AKT phosphorylation. During the 90 min incubation with OPN, PF-573228 (30 µM; Sigma-Aldrich), LY294002 (30 µM; Sigma-Aldrich) or PD98059 (30 µM; Cell Signaling, Danvers, MA) were added into the medium to inhibit focal adhesion kinase (FAK), phosphatidylinositol 3-kinase (PI3K) or mitogen-activated protein kinase kinase 1/2 (MEK1/2) activities, respectively, as previously described [42], [43].

### RNA Extraction, RT-PCR and qRT-PCR

Thirty blastocysts were pooled and total RNA extracted with the Picopure RNA Isolation Kit (Arcturus Bioscience, Mountain View, CA). Total RNAs from uterine tissue were prepared with the ISOGEN reagent (Nippon gene, Tokyo, Japan). Total RNAs from blastocysts or uterine tissues were reverse transcribed and used for PCR analyses. Reverse transcribed cDNAs were also used for qRT-PCR analyses. Real-time amplification of cDNAs was carried out with a LightCycler 480 (Roche, Mannheim, Germany) and FastStart Universal SYBR Green Master (Roche) according to the manufacturer’s instruction. All reactions were performed in duplicate and results analyzed with the Fit Point Method included in the LightCycler 480 Software (Roche). *Hprt* served as internal control. Primer sequences and positive control tissues used in the RT-PCR and qRT-PCR analyses are presented in Table S1.

### Immunohistochemistry, in situ Hybridization and Whole Mount Immunofluorescence

Immunostaining of uterine tissues were performed on 10 µm fresh frozen sections as previously described [44]. Rabbit anti-human OPN (1∶1000 dilution; catalog no. ab8448; Abcam) and rabbit anti-mouse FN (1∶200 dilution; catalog no. ab23750; Abcam) antibodies were used. Tissue sections were counter-stained with hematoxylin. Negative controls were performed by substituting primary antibodies with normal rabbit IgG. In situ hybridization for *Spp1* mRNA was performed on 10 µm fresh frozen sections as previously described [45]. Sense and antisense digoxigenin-labeled riboprobes for *Spp1* were used [46]. Hybridized probes were visualized with an alkaline phosphatase-coupled anti-digoxigenin antibody (Roche). For whole mount immunofluorescence of blastocysts, samples were fixed with 4% paraformaldehyde and permeabilized in PBS containing 0.1% Triton X-100. Blastocysts were incubated with rat anti-mouse integrin β1 (1∶50 dilution; catalog no. 550531; BD Pharmingen, Franklin Lakes, NJ), rabbit anti-human VCL (1∶100 dilution; catalog no. V4139; Sigma-Aldrich), rabbit anti-human OPN (1∶200 dilution; catalog no. ab8448; Abcam) or rabbit anti-mouse FN (1∶200 dilution; ab23750; Abcam) antibodies, then labeled with Allexa 568 or FITC. Phalloidin-Atto 488 (Sigma-Aldrich) and DAPI (Sigma-Aldrich) were used to label F-actin and nuclei. The labeled blastocysts were examined with a confocal laser-scanning microscope (FV500, Olympus).

### Western Blot Analysis

Ten blastocysts were pooled and lysed in 10 µl of RIPA buffer. Protein lysates (2.5 µl of buffer/lane) were resolved on 10% SDS-PAGE gels and transferred to nitrocellulose membranes. Rabbit anti-human VCL (1∶1000 dilution; catalog no. 550531; BD Pharmingen), rabbit anti-mouse total AKT (1∶1000 dilution; catalog no. 559028; BD Pharmingen), rabbit anti-mouse Phospho-AKT (threonine 308, 1∶1000 dilution; catalog no. 9275; Cell Signaling), and rabbit anti-mouse Phospho-AKT (serine 473, 1∶1000 dilution; catalog no. 9271; Cell Signaling) antibodies were used. Immunoreactive proteins were detected using ECL plus reagents (GE Healthcare, Waukesha, WI). Western blotting of β-actin served as a loading control (1∶1000 dilution; catalog no. ab1801; Abcam).

### Statistical Analyses

Statistical analyses were performed using the R statistical package (http://www.r-project.org/). All qRT-PCR results were analyzed by analyses of variance followed by Tukey contrasts tests.

## Supporting Information

Table S1 **Primer sequences and positive control tissues used for RT-PCR and qRT-PCR.**
(XLS)
